# The effect of regional citrate anti-coagulation on the coagulation system in critically ill patients receiving continuous renal replacement therapy for acute kidney injury - an observational cohort study

**DOI:** 10.1186/s12882-017-0718-z

**Published:** 2017-10-02

**Authors:** Richard Fisher, Katie Lei, Mike J. Mitchell, Gary W. Moore, Helen Dickie, Linda Tovey, Siobhan Crichton, Marlies Ostermann

**Affiliations:** 1 Guy’s & St Thomas’ NHS Foundation Trust, Department of Critical Care, London, SE1 9RT UK; 2 St Thomas’ Hospital, Molecular Haemostasis & Thrombosis, London, SE1 9RT UK; 3 St Thomas’ Hospital, Diagnostic Haemostasis & Thrombosis Laboratory, London, SE1 9RT UK; 4 MRC Clinical Trials Unit, University College London, London, WC2B 6NH UK; 5 King’s College London, Guy’s & St Thomas’ NHS Foundation Trust, Department of Critical Care, London, SE1 9RT UK

**Keywords:** Acute kidney injury, Citrate, Anticoagulation, Renal replacement therapy

## Abstract

**Background:**

Regional anticoagulation with citrate is the recommended first line treatment for patients receiving continuous renal replacement therapy (CRRT). There is wide variability in filter patency which may be due to differences in patient characteristics and local practice. It is also possible that citrate has effects on primary and secondary haemostasis, fibrinolysis and platelet function that are still unknown. The primary aim of the study is to describe the effect of citrate on coagulation and fibrinolysis pathways in both the patient and the haemodialysis circuit.

**Methods:**

The study will recruit 12 adult patients admitted to the intensive care unit, requiring CRRT with regional citrate anticoagulation for acute kidney injury. Patients with pre-existing thrombotic or bleeding tendencies will be excluded. Thrombin generation, clot lysis and platelet function will be measured at baseline and at 12, 24, 36, 48 and 72 h after commencing CRRT (from the patient and from the circuit). We will describe the evolution of parameters over time as well as the differences in parameters between the patient and the circuit.

**Discussion:**

The study will provide new data on the effects of citrate during continuous renal replacement therapy which is not currently available. We will minimise confounding factors through the use of tight exclusion criteria and accept that this will slow down recruitment. Depending on the results, we hope to incorporate the findings into existing clinical guidelines and clinical practice with the aim to prevent premature filter clotting and interruptions in treatment.

**Trial registration:**

The study was registered with clinicaltrials.gov on 10th June 2015 (NCT02486614).

## Background

**Fig. 1:**
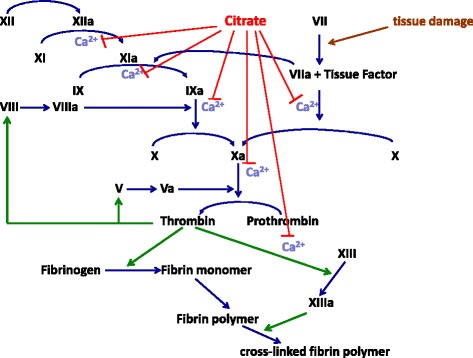
Known effects of citrate on clotting pathways

Citrate has emerged as the recommended first line anticoagulant for continuous renal replacement therapy (CRRT) in the Intensive Care Unit (ICU) based on its efficacy and safety profile [1]. It acts by chelating calcium and inhibiting the blood coagulation chemistry at several levels (Fig. 1). Several randomized controlled trials have shown significantly longer filter patency with citrate compared to anticoagulation with heparin [2]. However, the reported circuit life with citrate anticoagulation varies from a mean of 16 h to 125 h [2]. The exact reasons for this variation are not clear but are likely to be multifactorial.

Patient characteristics and vascular access problems are important risk factors for premature clotting. In fact, in patients with acute kidney injury (AKI) and sepsis, the coagulation/fibrinolysis balance is often deranged even prior to starting CRRT [3, 4]. A proportion of patients may also have an underlying medical condition that predisposes them to the formation of clots, such as Protein C deficiency or anti-phospholipid syndrome. In addition, no anticoagulant is effective at inhibiting all steps of the coagulation pathway completely. For instance, whilst citrate reduces thrombin generation by inhibiting reactions upstream, it does not impact all effects of thrombin (Fig. 1). It is also unknown whether citrate has any clinically important direct or indirect effects on fibrinolysis or platelet function [5–7]. Finally, not all events within the circuit are represented by systemic clotting results, and it has been argued that coagulation and fibrinolysis need to be studied separately in the patient and in the extracorporeal circuit [8]. We report our protocol to study the effects of citrate on coagulation and fibrinolysis pathways. (Version 1.1, 26 February 2014).

## Methods/design

### Aims

Our aim is to perform a detailed evaluation of the effects of citrate on the coagulation and fibrinolysis pathways during CRRT. The primary objective is to determine changes in primary haemostasis, coagulation and fibrinolysis parameters from baseline within the patient’s systemic circulation and the CRRT circuit over the course of 72 h of regional anticoagulation with citrate. The outcomes are parameters reflecting the coagulation and fibrinolysis pathways (see below).

### Design

We plan to conduct a prospective observational cohort study.

### Setting

The study is conducted in the critical care unit in a tertiary care centre in the United Kingdom.

### Patient selection

Patients with AKI in whom the treating team intend to commence on CRRT with citrate anti-coagulation will be screened against the inclusion and exclusion criteria. Patients meeting all of the inclusion criteria and none of the exclusion criteria will be recruited.

The inclusion criteria are:

- admitted to the critical care unit
- treating clinician plans to initiate CRRT with regional citrate anticoagulation
- age > 18 years
- expected to require CRRT for >72 h
- arterial or central venous catheter in situ to allow blood sampling

Exclusion criteria are:

- known pre-existing thrombotic tendency
- known pre-existing bleeding tendency
- disseminated Intravascular Coagulation (DIC)
- transfusion of any blood products in the 24 h before enrolment
- active bleeding (ie. needing blood transfusion) at time of enrolment
- haemoglobin at time of enrolment <75 g/L
- haematocrit at time of enrolment >0.55 L/L
- patient would refuse red blood cell transfusion (for example Jehova’s Witness)
- platelet count at time of enrolment <100 × 10^3^/μL
- treatment with any anticoagulant or antiplatelet agent at enrolment or within 7 days of enrolment with the exception of heparin or low molecular weight heparin for deep venous thromboembolism (DVT) prophylaxis
- intravenous heparin exposure within 4 h of commencing citrate anticoagulation
- malnourishment, ie. body mass index (BMI) <18.5 kg/m^2^ or unplanned weight loss >10% actual body weight (ABW) in preceding 6 months or BMI <20 kg/m^2^ and unplanned weight loss >5% ABW in preceding 6 months.
- CRRT prescribed for an indication other than acute kidney injury

### Sample size calculation

A formal sample size calculation was not possible due to lack of data in the literature. Following discussion with experts in the field, a sample size of 12 participants with complete data for 48–72 h was considered to be adequate to gain better understanding of coagulation and fibrinolysis during citrate based CRRT and to inform the design of future studies.

### Blood sampling

#### Baseline

The following blood tests will be performed at baseline, i.e. immediately prior to commencing CRRT: Full Blood Count (FBC), prothrombin time (PT), activated partial thromboplastin time ratio (APTTr), fibrinogen, D-Dimers, antithrombin activity, protein C activity, free protein S antigen, resistance to activated protein C (APCR) screening, homocysteine, PT 20210 mutation test, factor VIII activity, von Willebrand factor antigen, Dilute Russell’s Viper Venom Time (DRVVT), Dilute Activated Partial Thromboplastin Time (DAPTT), anticardiolipin antibodies, anti-beta 2 glycoprotein I antibodies, thrombin generation assay, clot lysis, prothrombin fragment 1 + 2, thrombin-antithrombin complexes (TAT) enzyme linked immunosobent assays (ELISA) and platelet function screening.

#### During CRRT

At 12, 24, 36, 48 and 72 h after commencing CRRT, blood samples will be taken from the systemic circulation and from the CRRT circuit to measure the following parameters: FBC, PT, APTTr, fibrinogen, D-Dimers, thrombin generation assay, clot lysis and platelet function screening.

### Laboratory tests

All laboratory tests except FBC will be conducted in the Haemostasis & Thrombosis Laboratories at Guy’s & St Thomas’ Hospital. Dade® Innovin® recombinant thromboplastin, Actin FS®, Thromboclotin®, Thrombin-Reagent®, Innovance D-dimer and HYPHEN BioMed Biophen FVIII:C chromogenic assay (Siemens Healthcare, Marburg, Germany) will be used on Sysmex CS2100i coagulation analysers (Sysmex UK, Milton Keynes, UK) for PT, APTT, Clauss fibrinogen, D-dimers and FVIII activity respectively. For lupus anticoagulant detection, DRVVT will employ Life Diagnostics LA Screen and LA Confirm reagents (Diagnostica Stago UK, Theale, UK), and DAPTT will use Stago PTT-LA (Diagnostica Stago) in the screen and addition of Bio/Data Corporation LA Confirmation Reagent (Alpha Labs, Eastleigh, UK) for the confirm. LA assays will be performed on a Sysmex 2000i analyser (Sysmex UK). Chromogenix Coamatic antithrombin, Coamatic protein C, Coatest APC Resistance, Coatest APCR resistance-V (Quadratech Diagnostic Ltd., Epsom, UK) and STA®-Liatest® Free protein S (Diagnostica Stago) will be used on Sysmex CS2000i analysers for antithrombin activity, protein C activity, phenotypic activated protein C resistance and free protein S antigen respectively. Inova Diagnostics QUANTA Lite® (ELISA) kits for IgG and IgM anticardiolipin and anti-β_2_ glycoprotein I antibodies (Werfen UK, Warrington, UK), Enzygnost® F1 + 2 and TAT will be performed on a Dynex DS2 ELISA analyser (Werfen UK) for anticardiolipin antibodies, anti-β_2_ glycoprotein I antibodies, prothrombin fragment 1 + 2 and thrombin-antithrombin complexes respectively. HemosIL VWF Antigen will be performed on an ACL TOP 500 coagulation analyser (Werfen UK) for von Willebrand factor antigen. Platelet function screening will be performed on a PFA-100 analyser (Sysmex UK) with collagen/ADP and collagen/epinephrine cartridges (Sysmex UK). Homocysteine will be assayed with Abbott ARCHITECT chemiluminescent microparticle assay (Abbott UK, Maidenhead, UK) on an Abbott ARCHITECT *i*2000SR immunoassay analyser (Abbott UK). The Thrombin Generation Assay will performed using a Technoclone kit (Technoclone, Vienna, Austria) on a Wallac Victor^3^ multilabel plate reader (Perkin Elmer, Turku, Finland). The Clot Lysis Assay will be performed as previously described [6] and read on a Wallac Victor^3^ multilabel plate reader (Perkin Elmer, Turku, Finland). The Prothrombin 20,210 polymorphism will be screened for using an in house developed allelic discrimination assay on an Applied Biosystems 7500 Real-Time PCR System (Applied Biosystems, Foster City, CA, USA).

### Baseline data collection

The following data will be collected: age, gender, ethnicity, admission diagnosis, comorbidities, biochemistry profile, C-reactive protein (CRP), Acute Physiology and Chronic Health Evaluation (APACHE) II score on admission and daily Sequential Organ Failure Assessment (SOFA) scores.

### Withdrawal criteria

Patients will be excluded from the study at their own request or the request of their legal representative. They will be excluded from further blood sampling if any of the following events occur during the 72 h sampling period:

- Development of Disseminated Intravascular Coagulation (DIC).
- Need for treatment with any systemic anticoagulant or antiplatelet agent with the exception of heparin or low molecular weight heparin for DVT prophylaxis
- Need for treatment with platelets or clotting factors

### Statistical analysis

The baseline characteristics will be summarised using appropriate descriptive statistics [i.e. frequency (percentage) for categorical data and mean (standard deviation) or median (interquartile range) for continuous data]. All coagulation and fibrinolysis parameters at baseline will be summarised as means (standard deviation) unless the distribution is highlight skewed, in which case median values (interquartile range) will be used.

Average coagulation and fibrinolysis parameters at each time point, and the mean (standard deviation) of the difference relative to baseline will be presented. The evolution of parameters will also be illustrated graphically by plotting the mean (with 95% confidence interval) against time points. Trajectories of individual patients over time will be shown graphically. Separate figures will be used to summarise data obtained from bloods taken from the patient and that from the CRRT circuit.

Repeated measures analysis of variance (ANOVA) (one for each of the parameters obtained from patient’s bloods and separately for those from the circuit), will be used to further explore changes in parameters over time. Where CRRT ends before 72 h patients will not have measurements taken at 72 h. The ANOVA will therefore include all measurements up to 48 h (unless all patients have complete data to 72 h, in which case the final time point will also be included). If a main effect of time is identified, post-hoc comparisons will be carried out to determine where differences lie.

Coagulation and fibrinolysis parameters from the patients’ blood will be plotted against the corresponding value obtained from blood drawn from the CRRT circuit. The mean (standard deviation) of the differences will be calculated overall and at each time point and Bland-Altman limits of agreement calculated.

## Discussion

To our best knowledge, this is the first study that will provide a detailed description of clotting and fibrinolysis processes before CRRT and the effects of citrate on coagulation pathways, fibrinolysis and platelet function during CRRT.

The strengths of this project are:

i) The project will provide data that are currently not available.
ii) By using very tight inclusion and exclusion criteria we are minimising confounding influences as much as possible.
iii) Serial blood samples will be taken from the systemic circulation and the CRRT circuit separately to differentiate between events in the patient and in the extracorporeal circuit.
iv) The results have potential to inform the design of future exploratory studies and intervention trials aimed a prolonging circuit life during CRRT and preventing unplanned interruptions in treatment.

We are aware of potential limitations and risks of the project which we will eliminate as much as possible.

i) As a result of very strict criteria for inclusion, exclusion and withdrawal and a relatively long sampling period, recruitment is likely to be slow.

It is possible that we identify medical problems through this research that may have implications for patients and their close relatives, for instance a genetic predisposition to clotting which was previously not known about. With approval by the Research Ethics Committee, we have put measures in place to make sure that affected patients and relatives will receive the necessary medical attention.

Depending on the findings of the study, we plan to incorporate the results into current guidelines and clinical practice and hope that premature filter clotting can be prevented and the delivery of CRRT improves.

## Abbreviations

ABW: actual body weight
AKI: Acute kidney injury
ANOVA: Repeated measures analysis of variance
APACHE: Acute Physiology and Chronic Health Evaluation
APCR: Resistance to activated protein C
APTTr: Activated partial thromboplastin time ratio
BMI: Body mass index
CRP: C-reactive protein
CRRT: Continuous renal replacement therapy
DAPTT: Dilute Activated Partial Thromboplastin Time (DAPTT)
DIC: Disseminated Intravascular Coagulation
DRVVT: Dilute Russell’s Viper Venom Time
DVT: Deep vein thrombosis
ELISA: Enzyme linked immunosobent assays
FBC: Full blood count
ICU: Intensive care unit
PT: Prothrombin time
SOFA: Sequential Organ Failure Assessment
TAT: Thrombin-antithrombin complexes
VWF: Von Willebrand factor
